# Evidence from stable-isotope labeling that catechol is an intermediate in salicylic acid catabolism in the flowers of *Silene latifolia* (white campion)

**DOI:** 10.1007/s00425-020-03410-5

**Published:** 2020-06-08

**Authors:** Kristen Van Gelder, Taylor Forrester, Tariq A. Akhtar

**Affiliations:** Department of Molecular and Cellular Biology, University of Guelph, Guelph, ON N1G 2W1 Canada

**Keywords:** Catabolism, Catechol, *O*-methyltransferase, Phytohormone, Salicylic acid

## Abstract

**Main conclusion:**

A stable isotope-assisted mass spectrometry-based platform was utilized to demonstrate that the plant hormone, salicylic acid, is catabolized to catechol, a widespread secondary plant compound.

**Abstract:**

The phytohormone salicylic acid (SA) plays a central role in the overall plant defense program, as well as various other aspects of plant growth and development. Although the biosynthetic steps toward SA are well documented, how SA is catabolized in plants remains poorly understood. Accordingly, in this study a series of stable isotope feeding experiments were performed with *Silene latifolia* (white campion) to explore possible routes of SA breakdown. *S. latifolia* flowers that were fed a solution of [^2^H_6_]-salicylic acid emitted the volatile and potent pollinator attractant, 1,2-dimethoxybenzene (veratrole), which contained the benzene ring-bound deuterium atoms. Extracts from these *S. latifolia* flowers revealed labeled catechol as a possible intermediate. After feeding flowers with [^2^H_6_]-catechol, the stable isotope was recovered in veratrole as well as its precursor, guaiacol. Addition of a trapping pool of guaiacol in combination with [^2^H_6_]-salicylic acid resulted in the accumulation of the label into catechol. Finally, we provide evidence for catechol *O*-methyltransferase enzyme activity in a population of *S. latifolia* that synthesizes veratrole from guaiacol. This activity was absent in non-veratrole emitting flowers. Taken together, these results imply the conversion of salicylic acid to veratrole in the following reaction sequence: salicylic acid > catechol > guaiacol > veratrole. This catabolic pathway for SA may also be embedded in other lineages of the plant kingdom, particularly those species which are known to accumulate catechol.

**Electronic supplementary material:**

The online version of this article (10.1007/s00425-020-03410-5) contains supplementary material, which is available to authorized users.

## Introduction

The plant hormone, salicylic acid (SA), plays a central role in the overall plant defense response against pathogens and mediates several other aspects of plant growth and development (Vlot et al. 2009; Dempsey et al. 2011; Klessig et al. 2018; Zhang and Li 2019). As observed with other plant hormones, the level of SA within tissues is known to be tightly regulated to ensure the proper function of defense and developmental programs (Zhang et al. 2010; Dempsey et al. 2011; Seguel et al. 2018). With over a half century of research into SA metabolism, the biosynthetic steps toward SA have been almost fully elucidated (Rekhter et al. 2019; Torrens-Spence et al. 2019). However, the catabolism of this essential plant hormone remains somewhat of an enigma.

In plants, there are two pathways that operate in SA biosynthesis: the isochorismate (IC) pathway in the plastid and the phenylalanine ammonia lyase (PAL) pathway within the cytosol (Dempsey et al. 2011). Depending on the particular species, one or the other pathway will typically predominate (Chen et al. 2009). In addition, each SA biosynthetic pathway can be preferentially triggered by specific abiotic and/or biotic factors (Gaffney et al. 1993; Delaney et al. 1994; Rivas-San Vicente and Plasencia 2011). While the precursors and intermediates of each pathway have been well defined through a combination of genetic and isotope-assisted labeling approaches in several plants (Yalpani et al. 1993; Ribnicky et al. 1998; Jarvis et al. 2000; Chong et al. 2001; Wildermuth 2006; Strawn et al. 2007), the genes encoding the final enzymatic steps for SA biosynthesis via the IC pathway were only recently identified in Arabidopsis (Rekhter et al. 2019; Torrens-Spence et al. 2019). The multi-drug and toxin extrusion transporter enhanced disease susceptibility5 (EDS5) first transports IC out of the plastid (Nawrath et al. 2002; Serrano et al. 2013), where it is conjugated to glutamate by the cytosolic amidotransferase, avrPphB susceptibile3 (PBS3), to isochorismoyl-glutamate (Jagadeeswaran et al. 2007; Lee et al. 2007; Nobuta et al. 2007; Rekhter et al. 2019). At present, it is believed that this intermediate is finally converted into SA via a combination of spontaneous non-enzymatic decomposition and the isochorismoyl-glutamate pyruvoyl-glutamate lyase activity of the enhanced Pseudomonas susceptibility (EPS1) protein (Zheng et al. 2009; Torrens-Spence et al. 2019). The genetic basis for SA biosynthesis via the PAL pathway is less well understood; however, isotopic labeling studies have demonstrated that it is derived from phenylalanine through *trans*-cinnamic acid and benzoic acid intermediates (El-Basyouni et al. 1964; Wildermuth et al. 2001; Chen et al. 2009; Dempsey et al. 2011).

**Fig. 1:**
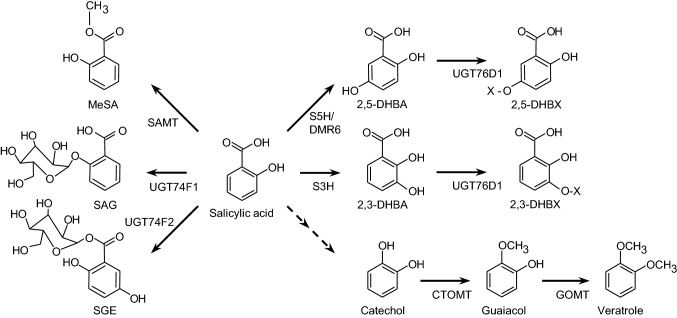
Routes of salicylic acid metabolism in plants. Dashed arrows indicate enzymatic steps that remain uncharacterized. Solid arrows indicate reactions for which plant enzymes have been identified. CTOMT, catechol *o*-methyltransferase; DHBA, dihydroxybenzoic acid; DHBX, dihydroxybenzoic glycoside; DMR6, Downy Mildew Resistant 6; GOMT, guaiacol o-methyltransferase; MeSA, methyl salicylate; S3H, salicylic acid 3-hydroxylase; S5H, salicylic acid 5-hydroxylase; SAMT, salicylic acid methyltransferase; SAG; salicylic acid glycoside; SGE, salicylic acid glucose ester; UGT, UDP-glucosyltransferase; X—glucose or xylose. UGT74F2 also catalyzes the formation of SAG and is commonly referred to as salicylic acid gluosyltransferase1 (SGT1)

Following its synthesis, the active pool of SA may be reduced via a series of modifications to its hydroxybenzoic acid backbone that form inactive storage forms of the hormone (Fig. 1). Glycosylation of SA to SA 2-O-*β*-d-glucose (SAG) or SA glucose ester (SGE) represent vacuolar storage pools (Dean et al. 2005; Dean and Delaney 2008; Song et al. 2008). Additional inactive forms of SA include 2,3- and 2,5-dihydroxybenzoic acid (2,3- and 2,5-DHBA) which are synthesized by salicylic acid 3-hydroxylase (S3H) and salicylic acid 5-hydroxylase (S5H/DMR6), respectively (Fig. 1; Zhang et al. 2013, 2017). 2,3- and 2,5-DHBA mainly exist as glycosides, either as O-*β*-d-glucosides or O-*β*-d-xylosides (Bartsch et al. 2010; Zhang et al. 2013), and are thought to be translocated to the vacuole for storage as well. SA can also be liberated from SAG by SA *β*-glucosidase (Seo et al. 1995), which ‘fine-tunes’ the bioavailability of SA. Finally, in both leaves and more commonly in floral organs, a methyl group from S-adenosine-L-methionine (SAM) can be added to the carboxyl group of SA by specific SA methyltransferases (Ross et al. 1999; Chen et al. 2003) to form methyl salicylate (MeSA). MeSA is a volatile compound emitted from flowers to attract pollinators. It has also been suggested that MeSA acts as a long-distance signal of SA-mediated defense programs (Chen et al. 2003; Park et al. 2008); however, this phenomenon has yet to be conclusively resolved (Attaran et al. 2009). No other routes for SA disposal or repurposing have been reported in planta.

Certain species of bacteria and fungi that can degrade aromatic hydrocarbons are known to convert SA into basic Krebs cycle intermediates (Ambrose et al. 2015). As a first step in this catabolic pathway, SA is oxidatively decarboxylated—the prototypical salicylate hydroxylase from *Pseudomonas putida*, NahG, catalyzes the hydroxylation and simultaneous decarboxylation of SA into catechol (Yamamoto et al. 1965). Transgenic plants that overexpress NahG are known to have lower levels of SA, accumulate high amounts of catechol, and are impaired in various defense responses against fungal, bacterial and viral pathogens (Gaffney et al. 1993; Delaney et al. 1994; van Wees and Glazebrook 2003). However, there have been no orthologs of NahG that have been identified in plants. It is noteworthy that several plant species are known to accumulate catechol (Towers et al. 1966; Morse et al. 2007; Mageroy et al. 2012), thus tempting speculation that analogous SA decarboxylative enzyme activity may also be present in such plants.

In this study, we explored SA catabolism using a stable isotope-assisted mass spectrometry platform that was recently developed to follow the fate of labeled SA through the flowers of the dioecious angiosperm, *Silene latifolia* (white campion). Similar to the aroma of many plants, the scent emitted by *S. latifolia* flowers comprises a mixture of volatile isoprenoids, phenylpropanoids and fatty-acid derivatives (Pichersky and Gershenzon 2002; Dötterl et al. 2005; Knudsen et al. 2006). Of particular note is 1,2-dimethoxybenzene (veratrole), a potent pollinator attractant which can account for up to 40% of the total floral bouquet of *S. latifolia* and which labeling studies previously indicated may in fact be derived from SA, through guaiacol, another volatile pollinator attractant (Dötterl et al. 2005, 2006; Akhtar and Pichersky 2013). Accordingly, we utilized this *S. latifolia* system to probe for possible intermediates in SA catabolism using a combination of stable isotope labeling, isotope trapping, and enzymatic assay approaches. Evidence is provided for a catabolic pathway for SA that involves catechol as a central intermediate.

## Materials and methods

### Chemicals and reagents

Deuterium-labeled 1,2-dihydroxybenzene-d6 (98% atom deuterium) and 2-hydroxybenzoic acid-d6 (98.8% atom deuterium) were from CDN isotopes (Pointe-Claire, QC, Canada). S-[Methyl-^14^C] adenosyl-L-methionine (2.146 GBq) was from PerkinElmer. Catechol, guaiacol, salicylic acid, 1,2-dihdroxybenzoic acid, and veratrole were obtained from Sigma-Aldrich (Oakville, ON, Canada). All other materials were obtained from Sigma-Aldrich or Fisher Scientific.

### Plant material and growth conditions

*Silene latifolia* seeds were obtained from a wild population originating from Blacksburg, VA, USA. Plants were grown in potting soil supplemented with Osmocote (Scotts) and maintained in growth chambers under a 16 h photoperiod (150 µmol m^−2^ s^−1^; mixed cool white and incandescent bulbs). Temperature was maintained at 23 °C during the light period and 18 °C during darkness. Relative humidity was kept at 60%. The plant material was identified and authenticated by Dr. Carole Ann Lacroix and a voucher specimen (No. 102507) was deposited at the Ontario Agricultural College Herbarium in Guelph, Ontario, Canada.

### Substrate feeding and volatile analysis

Fully developed male or female *S. latifolia* flowers were excised at the base of the calyx, placed in a 10 mL solution containing substrate and volatiles were collected by solid-phase microextraction (SPME) according to the method described by Akhtar and Pichersky (2013) with minor modifications. All substrates were supplied at a concentration of 10 mM and flowers were left in the solution under light for 1 h. The setup was then transferred to the dark for 1.5 h, followed by 20 min of volatile collection. Volatiles were desorbed from the SPME fiber and analyzed with an Agilent 7890A gas chromatography system coupled to an Agilent 5975C triple axis mass selective detector equipped with a Bruker BR-SWAX column (30-m length, 0.25 mm I.D., 0.50 μm phase thickness) using the following temperature program: 44 °C for 3.5 min, ramp of 5 °C min^−1^ to 200 °C, ramp of 70 °C min^−1^ to 250 °C, and hold for 1 min. The desorption was performed within the injector in splitless mode at 280 °C. Helium was used as the carrier gas at a rate of 1 mL min^−1^. The mass spectrometer was scanned from 50 to 550 *m/z* at a cycle of 1 s. Electron impact ionization at 70 eV was used with the source at 200 °C, while the transfer line was kept at 250 °C. The data were acquired after a 3 min solvent delay in positive ion mode and the assignment of each chromatographic peak was determined by searching the GC–MS mass spectral library (US National Institute of Standards and Technology, NIST 2011). Once the peaks were tentatively identified with the mass spectral library, the authentic standards were injected for positive identification in comparison with retention times and mass spectra.

### Catechol extraction and analysis by GC–MS/MS

*Silene latifolia* flowers (~ 200 mg) were flash frozen in liquid nitrogen and ground to a fine powder with a mortar and pestle. Catechol was extracted by adding five volumes of methanol:H_2_O (7:3, *v*/*v*) to the tissue powder, followed by rigorous shaking for 1 min. After incubation for 24 h at 4 °C, extracts were clarified by centrifugation at 12,000* g* for 5 min and the supernatant was dried *in vacuo*. Dried samples were silylated with 400µL of N-methyl-N-(trimethylsilyl)trifluoroacetamide (MSTFA) at 70 °C for 2 h and immediately analyzed with a Scion TQ GC–MS/MS (Bruker Daltonics Inc.) equipped with an Agilent DB-5MS column (30-m length, 0.25 mm I.D., 0.25 μm film thickness) using the following program: 80 °C for 2 min, ramp of 10 °C min^−1^ to 230 °C, ramp of 40 °C min^−1^ to 310 °C, and hold for 5 min. The inlet temperature was 280 °C and sample injection was performed in splitless mode. Helium was used as the carrier gas at a rate of 1 mL min^−1^. Mass spectral data were acquired in electron impact (EI)-positive ionization mode at 70 eV with selected reaction monitoring (SRM) using 2mTorr collision pressure and 30 eV for precursor ion fragmentation mode. The precursor and product ion transitions for catechol and deuterium-labeled catechol were established using authentic standards and were 254 to  > 73 m*/z* and 258 to  > 73 m*/z*, respectively.

### Protein extraction and CTOMT enzyme assays

Flash-frozen plant material was ground to a fine powder and protein was extracted in Buffer A [100 mM Tris–HCl, pH 7.5, 5 mM MgCl_2_, 10 mM *β*-mercaptoethanol and 10% glycerol (w/v)]. Soluble crude extracts were centrifuged at 4 °C and the supernatant was desalted on PD-10 columns (GE Healthcare) or using Sephadex G25 minicolumns (Helmerhorst and Stokes 1980) equilibrated in Buffer A. Protein concentration was determined by the method of Bradford (1976). Enzyme assays were conducted at room temperature and contained ~ 15 µg of desalted crude extract, 1 mM catechol, and 5 µM S-[methyl-^14^C]adenosyl-l-methionine (2.146 GBq) in a final volume of 50µL containing Buffer A. Reaction products were extracted with 200 µL of ethyl acetate and quantified using a scintillation counter (model LS 6500; Beckman). To identify reaction products, enzyme assays were conducted as described above with 200 µM S-adenosyl-L-methionine and analyzed by GC–MS according to the method used for volatile analysis.

## Results

### Stable isotope feeding points to catechol as a precursor in veratrole biosynthesis

**Fig. 2:**
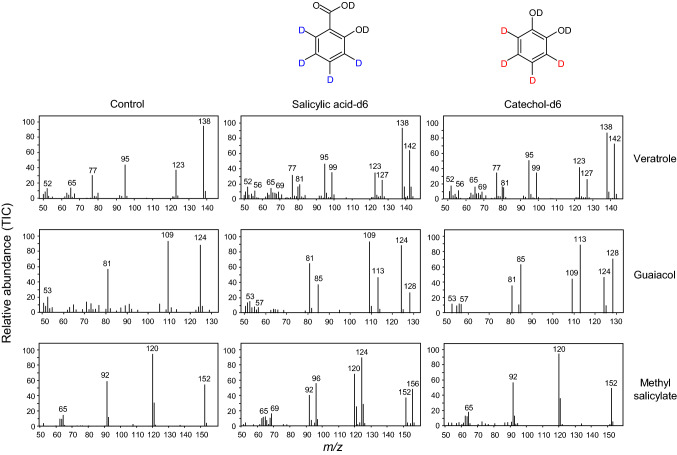
Stable isotope incorporation into *S. latifolia* volatiles. Detached flowers were fed water (control), solutions containing 10 mM [^2^H_6_]-catechol (CA-d6), or 10 mM [^2^H_6_]-salicylic acid (SA-d6) for 1.5 h in the dark and volatiles were then analyzed by GC–MS. The mass spectra (TIC) of veratrole, guaiacol, and methyl salicylate emitted from the flowers are shown. Note the 4 mass unit shift of the parent ions and mass spectral fragments of veratrole (*m/z* = 138), guaiacol (*m/z* = 124), and methyl salicylate (*m/z* = 152) upon feeding with CA-d6 or SA-d6. Mass spectra are representative of a minimum of six feeding experiments

*S. latifolia* is a dioecious angiosperm that emits an array of volatile compounds from both its male and female flowers (Jürgens et al. 2002; Dötterl et al. 2005). Among these emitted volatiles is the potent pollinator attractant known as veratrole (1,2-dimethoxybenzene), which is believed to be derived from salicylic acid based on stable isotope labeling studies (Akhtar and Pichersky 2013). Indeed, we confirmed that *S. latifolia* flowers that were fed a solution of deuterium-labeled [^2^H_6_]-salicylic acid (SA-d6) emit a portion of veratrole molecules with a 4-Da shift in the mass of the parent ion and its associated mass spectral fragments (Fig. 2), as originally demonstrated by Akhtar and Pichersky (2013). It should be noted that only four benzene ring-bound deuterium atoms remain on the SA-d6 label in planta, as the carboxyl and hydroxyl group readily ionize and undergo H/D back exchange when in solution, respectively. A similar 4-Da shift in the parent ions and associated mass spectral fragments of guaiacol and MeSA was also observed in the floral bouquet from these flowers, as previously demonstrated by Akhtar and Pichersky (2013; Fig. 2). While it was expected that the emitted MeSA would capture the fed label since SA is its immediate precursor, the incorporation of the SA-derived label into guaiacol was intriguing for two reasons. First, guaiacol is the immediate precursor to veratrole in plants that synthesize these two compounds (Gupta et al. 2012; Koeduka et al. 2016). Second, guaiacol is known to be derived from catechol in certain plants (Mageroy et al. 2012). While the origin of catechol in plants remains unclear, these labeling studies imply the involvement of catechol as a precursor to veratrole biosynthesis in the flowers of *S. latifolia*. Therefore, we formally tested this possibility by feeding flowers a solution of [^2^H_6_]-catechol (CA-d6) and then subsequently monitored the incorporation of the label into the volatiles that were released. A portion of both guaiacol and veratrole molecules that were emitted from these flowers exhibited a 4-Da shift in the mass of their parent ions and associated mass spectral fragments; no such shift in the mass of emitted MeSA from these flowers was observed (Fig. 2). Taken together, these labeling studies reveal that the benzene ring of veratrole and its immediate precursor, guaiacol, can be derived from either SA and/or catechol. These results, however, do not clarify where catechol fits, if at all, into the SA catabolic network.

### Isotope trapping implicates catechol as an intermediate in SA catabolism

**Fig. 3:**
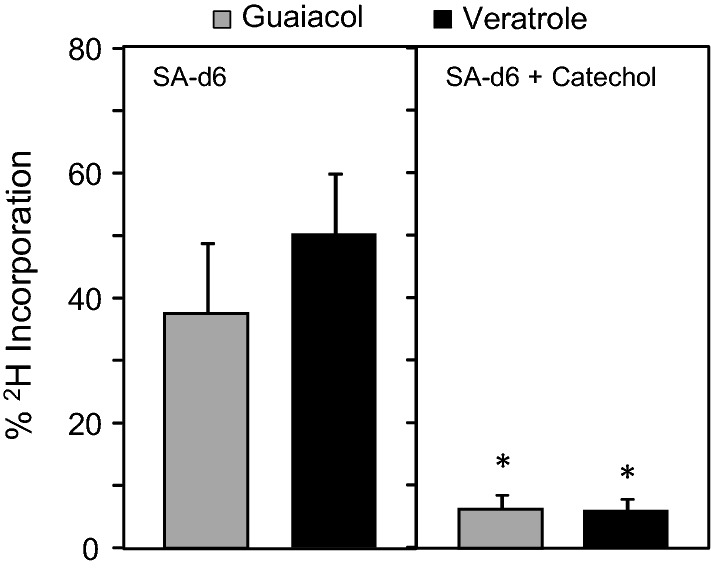
Stable isotope trapping of [^2^H_6_]-salicylic acid. Detached flowers were placed in solution containing 10 mM deuterium-labeled salicylic acid (SA-d6), with or without 10 mM unlabeled catechol. Volatiles were collected by solid-phase microextraction and analyzed by GC–MS. The incorporation of the stable isotope into the parent ion of guaiacol and veratrole is expressed as a percentage of the total for each compound. Data are the means ± SD of four separate feeding experiments and asterisks indicate a significant difference (*P* < 0.05) of isotope incorporation into guaiacol and veratrole between the two experiments

To clarify how the label from SA-d6 into veratrole proceeds and how catechol might partake in this pathway, a series of isotope trapping experiments were next performed. First, *S. latifolia* flowers were placed in a solution of SA-d6 with or without unlabeled catechol and the incorporation of the stable isotope label into both guaiacol and veratrole were determined. On average, approximately 45% of these emitted volatiles incorporated the deuterium label when fed SA-d6 alone; however, when unlabeled catechol was included in these feeding studies, the incorporation of the label into guaiacol and veratrole fell below 10% (Fig. 3). Together, these results suggest that catechol can trap the SA-d6 label from being incorporated into veratrole and potentially operate downstream from SA en route to the biosynthesis of veratrole in *S. latifolia* flowers.

**Fig. 4:**
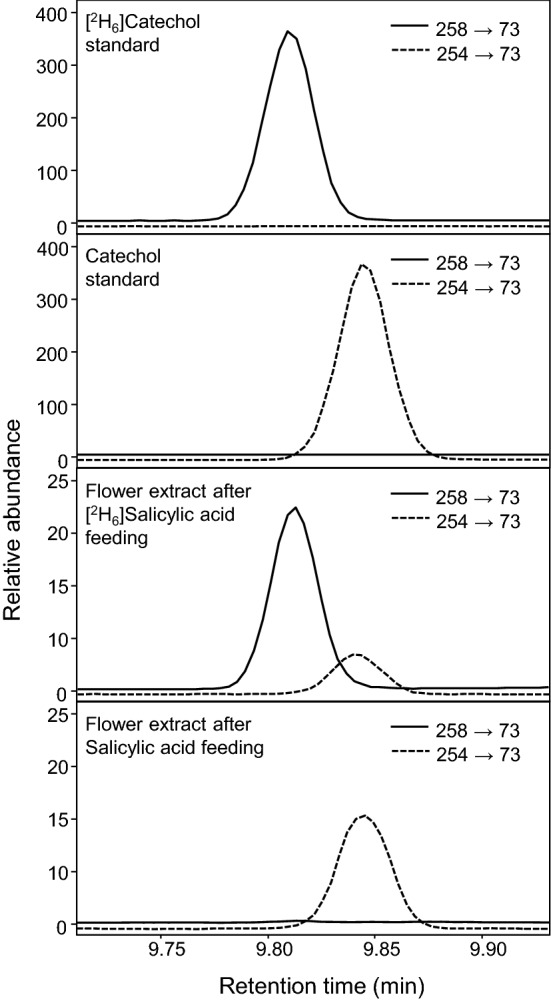
Evidence for the *in planta* conversion of salicylic acid to catechol. Soluble extracts from *S. latifolia* flowers that were fed a solution of deuterium-labeled [^2^H_6_]-salicylic acid or unlabeled salicylic acid were analyzed for the presence of deuterium-labeled catechol and catechol. A GC–MS/MS method with selected reaction monitoring was developed to monitor the mass transitions of 258–73 and 254–73 for labeled and unlabeled catechol, respectively. The top two panels illustrate the detection of authentic standards of labeled catechol and unlabeled catechol using this method. The bottom two panels illustrate the analysis of extracts from flower tissue that were fed the indicated compounds

To demonstrate that the label derived from SA-d6 could be converted into catechol, *in planta*, a tandem mass spectrometry method was developed to monitor labeled and unlabeled catechol in extracts from *S. latifolia* flowers that were accordingly fed the labeled substrate. Following derivatization of methanolic extracts from these flowers (via silylation), selected reaction monitoring was able to unambiguously detect mass transitions for labeled catechol (258–73) and unlabeled catechol (254–73) in these samples according to their comparison with authentically processed standards (Fig. 4, Fig. S1). In flowers that were fed unlabeled SA, only unlabeled catechol could be detected; however in extracts from those flowers that were fed SA-d6, both labeled and unlabeled catechol were present (Fig. 4). In the context of SA catabolism, these labeling studies provide evidence for the conversion of SA into catechol.

Next, a dose-dependent isotope trapping experiment was performed by providing the SA-d6 label to flowers along with increasing amounts of unlabeled guaiacol. Three general trends were observed in these flowers: first, the proportion of emitted veratrole molecules that contained the label steadily decreased as the dose of unlabeled guaiacol was increased; second, and conversely, the internal pool of labeled catechol increased in accordance with the amount of unlabeled guaiacol that was co-administered with the SA-d6 label to these flowers; third, a steady increase in the proportion of emitted MeSA containing the label was observed with increasing amounts of the unlabeled guaiacol that was administered (Table 1). To corroborate these observations, the SA-d6 label was then replaced with a solution of CA-d6 along with increasing amounts of unlabeled guaiacol and the incorporation of the isotope was monitored accordingly. In these flowers, the internal pool of labeled catechol progressively increased while the proportion of emitted veratrole containing the label steadily decreased with increasing doses of unlabeled guaiacol, respectively (Table 2). To summarize, the results of these labeling studies reveal the following: (1) the in vivo transfer of the SA-d6 label into veratrole can be inhibited by co-administration of either catechol or guaiacol, and that (2) the in vivo transfer of the CA-d6 label into veratrole can also be inhibited by guaiacol. Taken together, a linear catabolic pathway for SA is therefore implied, which first involves the conversion of SA into catechol followed by its conversion into guaiacol and then ultimately to veratrole (Fig. 1).

**Table 1:** Guaiacol as a trapping pool for [^2^H_6_] salicylic acid catabolism

Unlabeled Guaiacol Dose	[^2^H] Distribution		
	Methyl salicylate	Veratrole	Internal [^2^H_6_] Catechol
mM	% parent ion	% parent ion	ng g^−1^ fresh wt
0	43.20 ± 2.17	38.5 ± 6.06	0.46 ± 0.3
1	47.75 ± 8.84	28.40 ± 2.94	0.55 ± 0.19
2	50.40 ± 2.06	12.50 ± 0.69	0.90 ± 0.37
5	66.25 ± 2.86	5.37 ± 2.02	1.42 ± 0.63

Detached flowers were fed a solution containing 10 mM [^2^H_6_]-salicylic acid in combination with the indicated concentrations of guaiacol. The conversion of [^2^H_6_]-salicylic acid into methyl salicylate and veratrole was determined by the incorporation of ^2^H into their respective parent ions of methyl salicylate and veratrole were determined by GC–MS and is expressed as a percentage of the total. Data are the mean values ± SE from five independent feeding experiments

**Table 2:** Guaiacol prevents [^2^H_6_]-catechol incorporation into veratrole

Unlabeled Guaiacol dose	[^2^H] Incorporation in veratrole	Internal [^2^H_6_] catechol
mM	% parent ion	ng g^−1^ fresh wt
0	45.13 ± 6.71	4.54 ± 2.84
1	22.25 ± 3.41	12.73 ± 5.99
2	12.97 ± 2.37*	10.65 ± 4.42
5	9.33 ± 0.41*	22.85 ± 11.86

Detached flowers were fed a solution containing 10 mM [^2^H_6_]-catechol together with the indicated doses of guaiacol for a period of 2 h. Following the feeding period, volatiles emitted by the detached flowers were collected and the amount of ^2^H incorporated into the parent ion of veratrole was quantified by GC–MS and expressed as the percentage of the total. The internal amount of [^2^H_6_]-catechol in the same detached flowers was then extracted and quantified by GC–MS/MS and expressed on a fresh weight basis. Data are the mean values ± SE from six independent feeding experiments. Asterisks indicate a significant difference (*P* < 0.05) compared to flowers that were not provided guaiacol

### Catechol O-methyltransferase activity in the flowers of *S. latifolia*

**Fig. 5:**
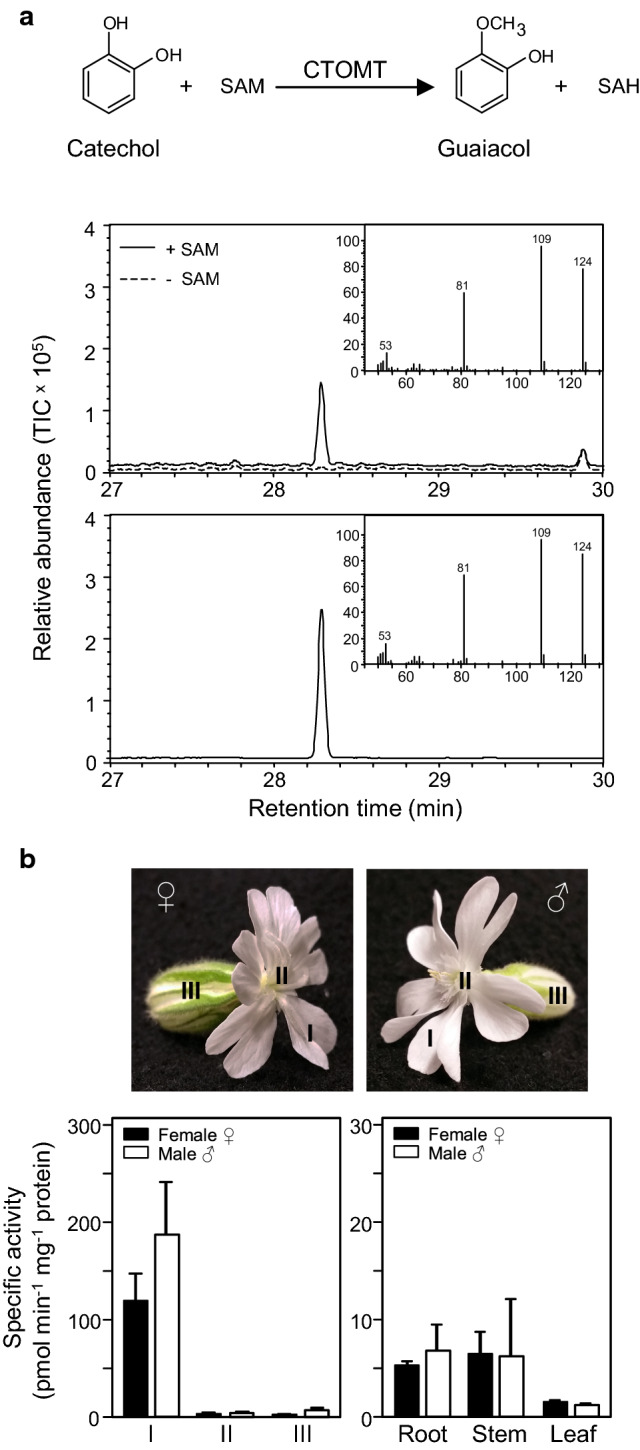
Catechol-*O*-methyltransferase enzyme activity in the flowers of *Silene latifolia*. **a** Evidence for the enzymatic conversion of catechol to guaiacol by an *S*-adenosyl-l-methionine (SAM)-dependent catechol-*O*-methyltransferase (CTMOT). Desalted crude flower extracts were assayed for CTOMT enzyme activity with or without SAM and the reaction products were analyzed by GC–MS. A single product peak was detected only in assays containing SAM (upper panel) whose mass spectra (inset) matched that of authentic guaiacol standard (lower panel). **b** Tissue distribution of CTOMT activity. Female and male flowers (*upper panel*) were dissected into petals (I), styles or stamens (II), and calyces (III), respectively, and enzyme activity was assayed in desalted crude extracts using 1 mM guaiacol and ^14^C-SAM (lower panel). Note that CTOMT enzyme activity in vegetative tissues (leaf, stem, and root) is several fold lower in comparison. Specific activity values are the means ± SD of three independent experiments using tissue pooled from three male or three female plants

Genetic and biochemical evidence for a guaiacol-*O*-methyltransferase (GOMT) in *S. latifolia* that catalyzes the conversion of guaiacol to veratrole lends support to an SA catabolic pathway in this plant species (Akhtar and Pichersky 2013; Gupta et al. 2012). To further explore this line of inquiry, we next surveyed *S. latifolia* for catechol-*O*-methyltransferase (CTOMT) enzyme activity, which would be responsible for converting catechol into guaiacol and would theoretically represent the penultimate step in SA catabolism, according to the previously described in planta labeling studies. As a precedent for this analysis, a CTOMT was previously established to be responsible for guaiacol biosynthesis in tomato fruit, where it serves as an important factor in governing aroma, flavor, and fruit quality (Mageroy et al. 2012). We first assayed for CTOMT activity in *S. latifolia* flowers by providing de-salted protein extracts with catechol and the universal methyl donor, *S*-adenosyl-L-methionine (SAM). The in vitro reaction products from these assays were then analyzed by GC–MS and shown to include a compound whose retention time and mass spectra matched that of an authentic guaiacol standard; assays without SAM were devoid of this enzymatic product and we found no evidence for non-enzymatic methylation of catechol (Fig. 5a; Fig. S2). We next analyzed CTOMT enzyme activity in a variety of *S. latifolia* tissues—male and female flowers were accordingly dissected into their reproductive organs and vegetative tissues. CTOMT enzyme activity was mostly concentrated in the petal tissue of both male and female flowers with minimal activity in the calyx, styles and stamens and minimal enzyme activity was found in non-floral tissues (Fig. 5b). It should be noted that only flower tissues are known to produce substantial amounts of guaiacol and veratrole, so the absence of significant CTOMT activity in non-floral tissues was therefore not surprising.

### The evolution of floral volatiles in *S. latifolia*

**Fig. 6:**
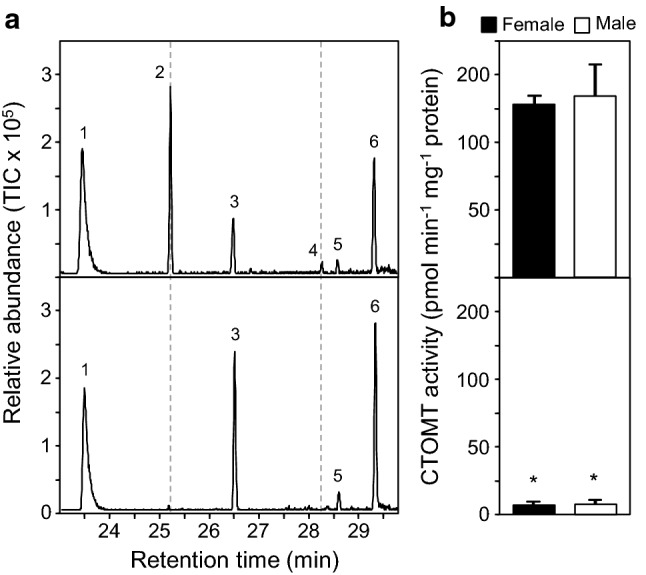
Veratrole emission depends on the expression of CTOMT. **a** Representative GC–MS traces of the volatile compounds emitted from flowers that produce veratrole and guaiacol (upper panel) and those that do not (lower panel).The missing veratrole (2) and guaiacol (4) peaks in the lower panel are indicated with dashed lines. The other peaks are indicated by the following numbers: (1) phenylacetaldehyde, (3) methyl salicylate, (5) benzyl alcohol, and (6) phenylethanol. **b** Total CTOMT enzyme activity in crude flower tissue extracts from veratrole and guaiacol emitters and non-emitters. Data are the mean values ± SE obtained from three independent extractions of pooled tissue from the corresponding groups. Asterisks (*) indicate statistical significance (*P* > 0.05)

Since the accidental introduction of *S. latifolia* to North America approximately 200 years ago, there have been a series of phenotypic changes within this population (Blair and Wolfe 2004). One such post-introduction evolutionary change has been the constitution of its floral bouquet, whereby many North American plants do not emit veratrole and/or other volatiles (Dötterl et al. 2005; Dötterl and Jürgens 2005; Akhtar and Pichersky 2013). Throughout the course of our analysis, we identified several plants (both male and female) which did not emit guaiacol and veratrole among this North American population (Fig. 6a). We predicted that a lack of CTMOT activity in these plants could account for this floral volatile phenotype, so enzymatic assays (as outlined above) were therefore conducted with emitters and non-emitters of both guaiacol and veratrole. Crude protein extracts from emitters of guaiacol and veratrole that were supplied catechol and [^14^C]-SAM readily produced labeled guaiacol; however, non-emitters completely lacked this CTOMT enzyme activity (Fig. 6b). These results lend genetic support for the role of CTOMT in both guaiacol and veratrole production and also highlight a unique resource to further study SA catabolism in *S. latifolia*, a point of discussion that we return to below.

## Discussion

Evidence is provided in this study for an in planta catabolic pathway for the essential plant hormone, salicylic acid (SA). Using flowers from *S. latifolia*, a series of stable isotope feeding and isotope trapping experiments were employed to demonstrate that SA can be converted into catechol, a widespread phenolic compound that appears to accumulate in several species throughout the plant kingdom—examples of catechol accumulation have been noted in several monocots and among members of the core eudicots (including individuals from the Rosids, Asterids, and Caryophyllales), as well as in some basal angiosperms (Tomazsewski 1960; Vázqueza et al. 1968; Yoshi-Stark et al. 2003; Yang et al. 2004; Morse et al. 2007; Mageroy et al. 2012). If the origin of catechol within these plants is also due, even in part, to SA catabolism, it raises two important questions: First, how is SA converted into catechol, and second, how do plants dispose of catechol?

The conversion of SA into catechol was first reported in *P. putida*, and subsequently in other related bacteria as well as in fungi (Yamamoto et al. 1965; Rabe et al. 2013; Ambrose et al. 2015; Rocheleau et al. 2019). In these organisms, SA is oxidatively decarboxylated directly into catechol by enzymes that belong to the flavin-dependent monooxygenase superfamily. The most studied and prototypical member from bacteria, NahG, utilizes FAD and NADPH as cofactors to regiospecifically replace the carboxyl group on SA with a hydroxyl moiety on the *ortho* position relative to the phenol group (Ambrose et al. 2015; Costa et al. 2019). Results from our stable isotope feeding experiments indeed support the notion of an analogous enzymatic conversion of SA directly into catechol, in planta. To date, however, an orthologous activity has yet to be ascribed to any single gene product in plants. Perhaps complicating the search for a bona fide FAD-dependent ‘salicylate-1-monooxygenase’ in plants is that the flavin-containing monooxygenase (FMO) family is large; Arabidopsis is predicted to have at least 29 FMO members (Schlaich 2007). Moreover, these enzymes are known to oxidize a diverse range of substrates, and while some plant FMOs possess narrow substrate specificity, it is unclear whether a strict relationship between sequence similarity and substrate preference exists for this family of enzymes (Hansen et al. 2007; Schlaich 2007; Dai et al. 2013; Huijbers et al. 2014; Kong et al. 2016; Hartmann et al. 2018). In addition, some members of the FMO superfamily are known to require a unique suite of partner proteins during the reduction step of their flavin cofactor (Ellis 2010). That being said, it cannot be ruled out that plants may utilize a different class of oxygenase-type enzymes altogether to catalyze the regiospecific decarboxylation and ortho-hydroxylation of SA into catechol.

In bacteria and fungi, catechol is a central intermediate in the ortho- and meta-cleavage pathways which operate to degrade a wide variety of aromatic substrates (including SA) into simple Krebs cycle constituents (Hamzah and Al-Baharna 1994; Cao et al. 2008; Wadke et al. 2016). For example, among many *Pseudomonas* sp., catechol is cleaved at the ortho position by catechol 1,2-dioxygenase to produce succinate and acetyl-CoA or by catechol 2,3-dioxygenase at the *meta* position to yield acetaldehyde and pyruvate (Loh and Chua 2002; Cao et al. 2008). Interestingly, SA is not the only phytohormone that can be used as a carbon source by these species and other related rhizobacterium—many can also convert the widespread plant hormone, indole-3-acetic acid (IAA), into catechol which is then processed through the same ortho- and meta-cleavage pathways (Donoso et al. 2016). Whether a similar avenue of aromatic catabolism has co-evolved within higher plants is not yet known. Nevertheless, there is some indication that the *meta*-cleavage pathway may be present in certain species of algae (Semple and Cain 1996) and it is well established that several plants have the capacity to convert simple phenolic compounds into CO_2_ (Tateoka 1970; Ellis 1971, 1977; Prasad and Ellis 1978), thereby tempting further inquiry into aromatic catabolism in higher plants.

It is generally thought that the active pools of most phytohormones, such as SA, are regulated by enzymatically modifying the parent molecule into storage forms by way of methylation, hydroxylation, glycosylation, or by other means (Seo et al. 2001; Ljung et al. 2002; Jin et al. 2013). Our data offer a new perspective, in that plants, such as *S. latifolia*, may possess a way of repurposing SA (and perhaps other aromatic metabolites) via a catabolic network that involves catechol as a central intermediate. In the case of *S. latifolia*, SA appears to be converted into catechol, which is then funneled into the synthesis of two potent pollinator attractants, namely guaiacol and veratrole. In tomato fruit, guaiacol synthesis from catechol also appears to have evolved as an important flavor-associated volatile that might influence the efficiency of seed dispersal (Mageroy et al. 2012). But what about plants that do not synthesize guaiacol or veratrole? The North American population of *S. latifolia* offers a tractable system to explore such catabolism, as this population appears to lack the ability to release SA catabolites in the form of volatile compounds (Akhtar and Pichersky 2013). Moving forward, an approach that combines the genetics of this unique population of plants with stable isotope labeling may serve as a useful tool to elucidate novel pathways in aromatic compound catabolism that have evolved in plants, akin to their bacterial ancestors.

### *Author contribution statement*

TAA conceived and designed the research. KVG, TF and TAA conducted the experiments. KVG and TAA analyzed the data and wrote the manuscript. All authors read and approved the manuscript.

## Electronic supplementary material

Below is the link to the electronic supplementary material.

Supplementary file1 (PPTX 64 kb)

Supplementary file2 (PPTX 64 kb)

## Abbreviations

CA-d6: [^2^H_6_]-Catechol
CTOMT: Catechol *O*-methyltransferase
MeSA: Methyl salicylate
SA: Salicylic acid
SA-d6: [^2^H_6_]-Salicylic acid
