# Supplementary material for: Evidence from stable-isotope labeling that catechol is an intermediate in salicylic acid catabolism in the flowers of Silene latifolia (white campion)
Source: Planta. 2020 Jun 8;252(1):3. doi: 10.1007/s00425-020-03410-5 (PMC7280317; doi:10.1007/s00425-020-03410-5)
Supplement: Supplementary file 1 — Supplementary file1 (PPTX 64 kb) [file 425_2020_3410_MOESM1_ESM.pptx]

## Slide 1
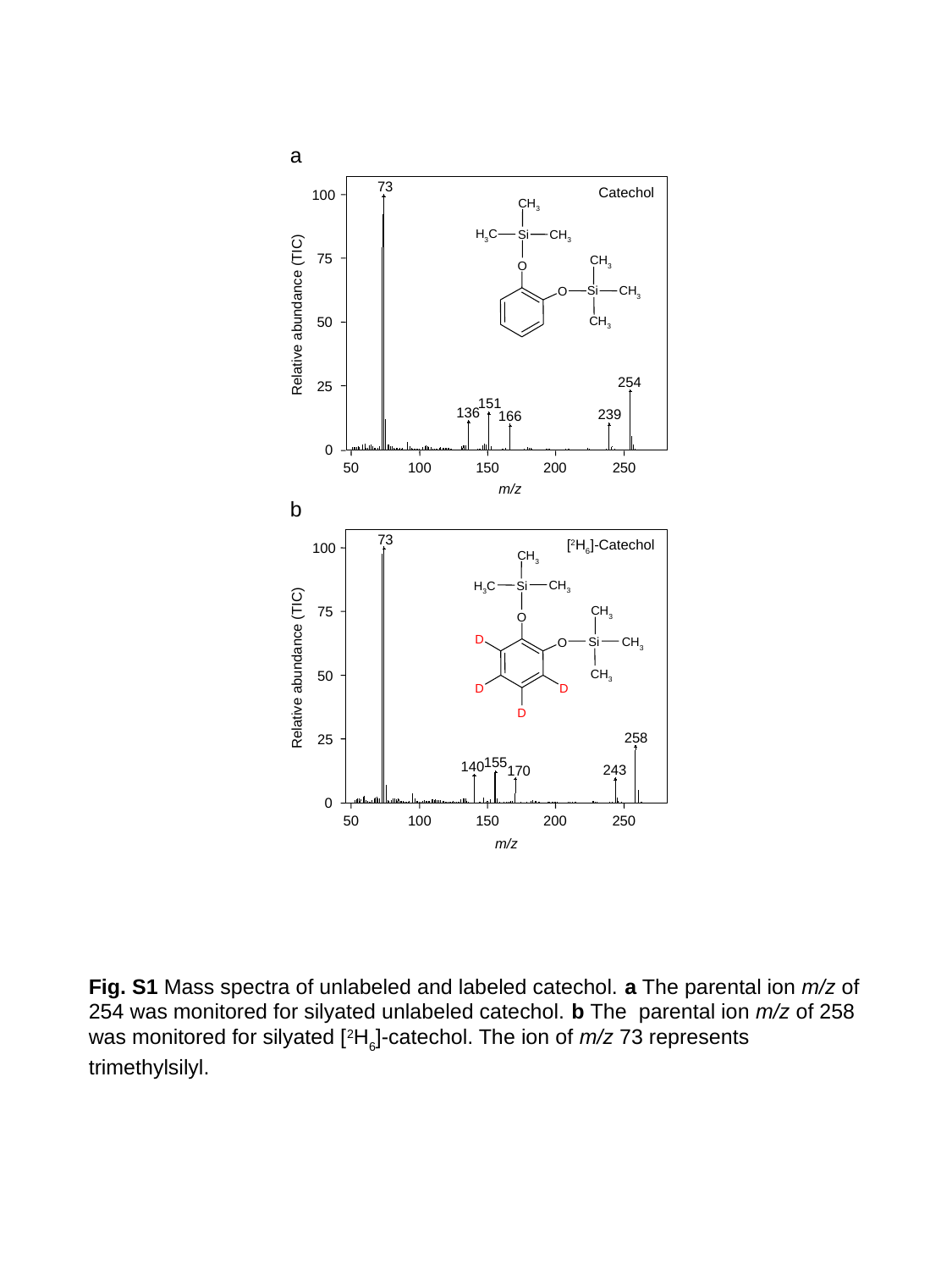

a
Catechol
 73
100
75
Relative abundance (TIC)
50
 254
25
 151
 136
 239
 166
0
50
100
150
200
250
m/z
CH3
3
H3C
CH3
Si
CH3
O
Si
CH3
O
CH3
b
[2H6]-Catechol
 73
100
75
Relative abundance (TIC)
50
 258
25
 155
 140
 243
 170
0
50
100
150
200
250
m/z
CH3
CH3
H3C
Si
CH3
O
D
Si
CH3
O
CH3
D
D
D
Fig. S1 Mass spectra of unlabeled and labeled catechol. a The parental ion m/z of 254 was monitored for silyated unlabeled catechol. b The parental ion m/z of 258 was monitored for silyated [2H6]-catechol. The ion of m/z 73 represents trimethylsilyl.
