# Supplementary material for: Evidence from stable-isotope labeling that catechol is an intermediate in salicylic acid catabolism in the flowers of Silene latifolia (white campion)
Source: Planta. 2020 Jun 8;252(1):3. doi: 10.1007/s00425-020-03410-5 (PMC7280317; doi:10.1007/s00425-020-03410-5)
Supplement: Supplementary file 2 — Supplementary file2 (PPTX 64 kb) [file 425_2020_3410_MOESM2_ESM.pptx]

## Slide 1
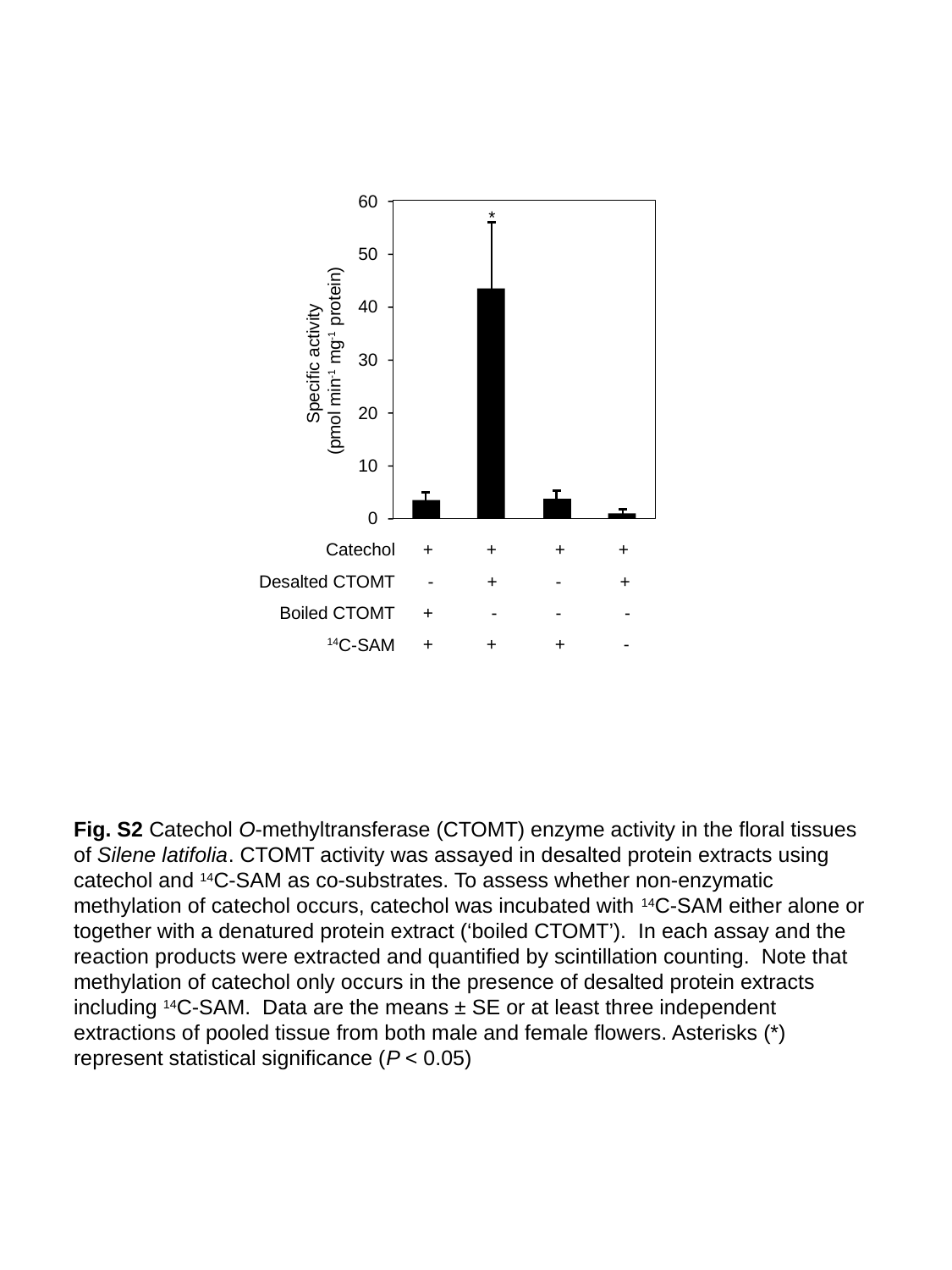

60
*
50
40
Specific activity
(pmol min-1 mg-1 protein)
30
20
10
0
 + + + +
 - + - +
 + - - -
 + + + -
Catechol
 Desalted CTOMT
Boiled CTOMT
14C-SAM
Fig. S2 Catechol O-methyltransferase (CTOMT) enzyme activity in the floral tissues of Silene latifolia. CTOMT activity was assayed in desalted protein extracts using catechol and 14C-SAM as co-substrates. To assess whether non-enzymatic methylation of catechol occurs, catechol was incubated with 14C-SAM either alone or together with a denatured protein extract (‘boiled CTOMT’). In each assay and the reaction products were extracted and quantified by scintillation counting. Note that methylation of catechol only occurs in the presence of desalted protein extracts including 14C-SAM. Data are the means ± SE or at least three independent extractions of pooled tissue from both male and female flowers. Asterisks (*) represent statistical significance (P < 0.05)
